# The anti-miR21 antagomir, a therapeutic tool for colorectal cancer, has a potential synergistic effect by perturbing an angiogenesis-associated miR30

**DOI:** 10.3389/fgene.2013.00301

**Published:** 2014-01-02

**Authors:** Min-Sun Song, John J. Rossi

**Affiliations:** ^1^Department of Molecular and Cellular Biology, Beckman Research Institute of City of HopeCity of Hope, Duarte, CA, USA; ^2^Irell and Manella Graduate School of Biological Sciences, Beckman Research Institute of City of HopeCity of Hope, Duarte, CA, USA

**Keywords:** microRNA, anti-miR21, perturbation, siRNA, colon cancer, angiogenesis

## Abstract

Colon cancer has the third highest incidence and mortality among cancers in the United States. MicroRNA-21 (miR21) has been described as an oncomir that is highly overexpressed in tumor tissue from colorectal cancer. Recent studies showed that silencing of miR21 through use of a miR21 inhibitor (anti-miR21) affected viability, apoptosis and the cell cycle in colon cancer cells. We identified an anti-miR21 that targets miR21 to inhibit genes by both post-transcriptional gene silencing and transcriptional gene silencing in the cytoplasm and nucleus, respectively. Overexpression of anti-miR21 in colon cancer cells caused changes in miRNA expression levels. We found that treatment with anti-miR21 down-regulated expression of miR30, which is involved in angiogenesis. In an *in vitro* angiogenesis assay, network formation induced by an angiogenesis activator was reduced upon treatment with anti-miR21. Sequence analysis of anti-miR21 and pri-miR30 revealed homology between anti-miR21 and the 3′ end of pri-miR30, suggesting that anti-miR21 may bind to pri-miR30 and block processing of the miRNA processing. These results suggest anti-miR21 has a role not only in tumor growth but also in angiogenesis. Therefore, treatment with the anti-miR21 antagomir may have a synergistic effect mediated through suppression of miR30.

## INTRODUCTION

Colorectal cancer (CRC) is a major challenge worldwide (Parkin et al., 2005) and is the third leading cause of cancer-related death in the United States (Kemp et al., 2004). The incidence of CRC has been increasing for decades, and although screening for CRC holds the promise of shifting the distribution of detected cancers toward those in earlier stages (Gross et al., 2006), surgery is still a cornerstone of CRC treatment. However, a considerable fraction of CRC patients treated with surgery alone will experience tumor recurrence. Therefore surgery is usually accompanied by adjuvant chemotherapy. Patients with Stage II CRC (no lymph node metastases or distant metastases) represent about 25% of all cases and have a 5-year overall survival (OS) of 73–85%, when surgically respected (O’Connell, 2004). Many efforts have been made to find new therapeutic adjuvant treatments.

MicroRNAs (miRNAs), which are small, noncoding, single-stranded RNAs that are usually 20–22 nucleotides in length, have been shown to play a role in CRC (Peters and Meister, 2007; Rana, 2007). miRNAs control gene expression by inducing the degradation or inhibiting the translation of target mRNAs by binding to complementary sequences in the 3′-untranslated region (3′ UTR) (Long and Lahiri, 2011; Zhang et al., 2011). This 3′ binding inhibits ribosome function, causing decapping of the capped 5′ end, deadenylation of the poly(A) tail and degradation of the target mRNA (Filipowicz et al., 2008). In addition to being important regulators of many biological processes such as the cell cycle, apoptosis, proliferation, or invasiveness, miRNAs can also affect the efficacy of anticancer therapies (Hanahan and Weinberg, 2000). Recent studies indicate that several miRNAs are differently expressed in normal and neoplastic colon tissues and they can be used to distinguish colon cancers relative to the histopathologic, prognostic, and predictive characteristics of the tumors (Hanahan and Weinberg, 2000; Slaby et al., 2007, 2009; Schetter et al., 2008; Chang et al., 2011).

MicroRNA-21 (miR21) is one of the miRNAs that are frequently overexpressed in CRC (Slaby et al., 2007; Schetter et al., 2008; Nielsen et al., 2011) and is, therefore, considered an onco-miRNA. Several studies have shown an association between elevated levels of miR21 and the downregulation of tumor suppressor genes, including programmed cell death 4 (PDCD4), tissue inhibitor of metalloproteinase 3 (TIMP3; Selaru et al., 2009), phosphatase and tensin homolog (PTEN; Wang et al., 2011), tropomyosin 1 (TPM1; Zhu et al., 2007), reversion-inducing cysteine-rich protein (RECK; Ziyan et al., 2011), ras homolog gene family member B (Liu et al., 2011), and maspin (Torres et al., 2011). This has led to miR21 being considered a promising therapeutic target for treating CRC. As an approach toward inhibiting miR21, we designed an anti-miR21 antagomir (complementary to the miR21^*^ sequence) that could inhibit miR21 (miRBase accession number: MIMAT0004494; Castanotto et al., 2007).

There is also mounting evidence that small RNAs, including miRNAs, play important roles in the nucleus and that some miRNAs, after being exported and post-transciptionally processed in the cytoplasm, then return to the nucleus (Politz et al., 2009). The nuclear sites and the functional significance of these nuclear-returning miRNAs are not known.

Although several research groups are focused on defining the effects of anti-miR21 in blocking carcinogenesis, its effects in CRC cells have not yet been determined. We sought to decipher the mechanism of action of anti-miR21 in CRC cells by quantifying the expression of various miRNAs and siRNAs and determining the localization of miR21 and other miRNAs within the cell. We found that anti-miR21 blocked the function of miR21 by acting within the nucleus to prevent miR21 from binding to the 3′ UTR of an EGFP target gene. Moreover, anti-mir21 perturbed the levels of miR30, another endogenous, CRC-associated miRNA that is unrelated to miR21 and involved in angiogenesis.

## MATERIALS AND METHODS

### CELL CULTURE STUDIES

Human CRC HCT116 cells were grown in Dulbecco’s modified Eagle’s medium (DMEM) supplemented with 10% fetal bovine serum (FBS), 100 units/ml penicillin, and 100 μg/ml streptomycin at 37°C in 5% CO_2_. The pcDNA4/GFPmir21 (Meister et al., 2004b) reporter has been previously described (Castanotto et al., 2007). One day before transfection, 1 × 10^6^ HCT116 cells, stably expressing pcDNA4/GFPmir21, were transfected with 100 nM anti-miR21 (5′-ACGGCAACACCAGUCGAUGGGCUGU-3′) using Lipofectamine 2000 according to the manufacturer’s protocol for six-well plates. EGFP expression was analyzed and documented using a Nikon eclipse TE2000-S fluorescence microscope and provided software.

Human umbilical vein endothelial cells (HUVEC2; BD Bioscience) were grown in EGM^TM^-2-MV bullet kit^TM^ (Lonza) at 37°C in 5% CO_2_ and used in experiments before passage five. Cy3-labeled anti-miR21 was transfected into HUVEC2 cells with RNAiMAX (50 nM, Invitrogen). Cy3-labeled siRNA was used as a negative control. Angiogenesis assays were performed with an *in vitro* angiogenesis assay kit (Abcam), according the manufacturer’s instructions.

### DETECTION OF miRNAs

TaqMan miRNA assays (Applied Biosystems) were used to quantify miR21, miR20, miR28, and miR30 in CRC cells. Briefly, cDNA synthesis was carried out using the TaqMan MicroRNA reverse transcription kit (Applied Biosystems). The miRNA reverse transcription-PCR primers for miR21, miR20, miR28, and miR30-5p, as well as the endogenous control 18s RNA, were purchased from Applied Biosystems. Real-time qRT-PCR analysis was performed using a CFX96^TM^ Real-time PCR System (Bio-Rad). The PCR mix contained TaqMan 2× Universal PCR Master Mix and was incubated as follows: 95°C for 10 min followed by 95°C for 15 s, 60°C for 60 s for up to 40 cycles. 18s RNA was used as the internal standard to normalize miRNA expression. The relative quantity (RQ) of different miRNAs was calculated as RQ = 2 - ΔΔCt.

### SMALL RNA DEEP SEQUENCING

HCT116 cells were grown to 70–80% confluence in DMEM in 10-cm dishes 1 day prior to transfection. Cells were transfected with 50 nM small RNAs through Lipofectamine 2000 methods (Invitrogen). At 48 h post-transfection, total RNAs were isolated with TriZol reagent (Invitrogen). RNA was resuspended in water (6 μl) and the entire volume was added to TruSeq Small RNA Sample reagents (Illumina). Samples were amplified by 15 cycles of PCR, clustered in a single read v3 flow cell, and deep sequenced for small RNA on a HiSeq 2000.

### BIOINFORMATICS

All analysis was performed using the R statistical environment and Bioconductor packages “Biostrings” and “ShortRead” (Morgan et al., 2009). The sequences generated from Illumina Pipeline v1.6 were matched to siRNA sense and anti-sense sequences through a seed-and-growth algorithm. First, sequences were searched for a siRNA seed sequence of 16 nucleotides after removing the 3′-adapter through the Bioconductor package “ShortRead.” For example, for an siRNA sequence of 23 nucleotides, the Illumina sequences were searched against eight seeds (i.e., sub-sequences from bases 1–16, 2–17, and so on of the original siRNA sequence). The matched sequences were then searched against the siRNA sequence with one base added to the 5′-end, one at a time until the sequence no longer perfectly matched the siRNA subsequence. The same procedure was used to grow the 3′-end of the matching sequence. When the growth steps were finished, the additional bases at either end of the sequence were considered as 5′ and 3′ that do not match the siRNA. The final matched sequences were reported by their relative start and end positions on the siRNA sequence, the non-matching bases at their ends, and the number of occurrences.

This set of sequences was then aligned along with each siRNA reference sequence using the ClustalX2 multiple alignment tool (Larkin et al., 2007), not allowing gaps. The multiple aligned sequences were visualized and exported using JalView (Waterhouse et al., 2009).

### CELL PROLIFERATION

Cells were seeded into 96-well microculture plates (15,000 cells/well) and transfected with 5 nM small RNAs or control siRNA through Lipofectamine 2000 methods (Invitrogen). After 24 h incubation, CellTiter 96 Aqueous One Solution Reagent (20 μl; MTS; Promega) was added to each well (into 100 μl of cell suspension). The plate was then incubated for 2 h at 37°C. MTS assay was used to measure cell viability. Specifically, the absorbance of soluble formazan produced by cellular reduction of MTS was measured at 490 nm. Values for the experimental conditions were normalized to a control value of 100 for each experiment.

### STATISTICAL ANALYSIS

All statistical analyzes were performed using a two-tailed unpaired Student’s t-test.

## RESULTS

### EFFICIENCY OF ANTI-miR21 (ANTAGOMIR)

**FIGURE 1:**
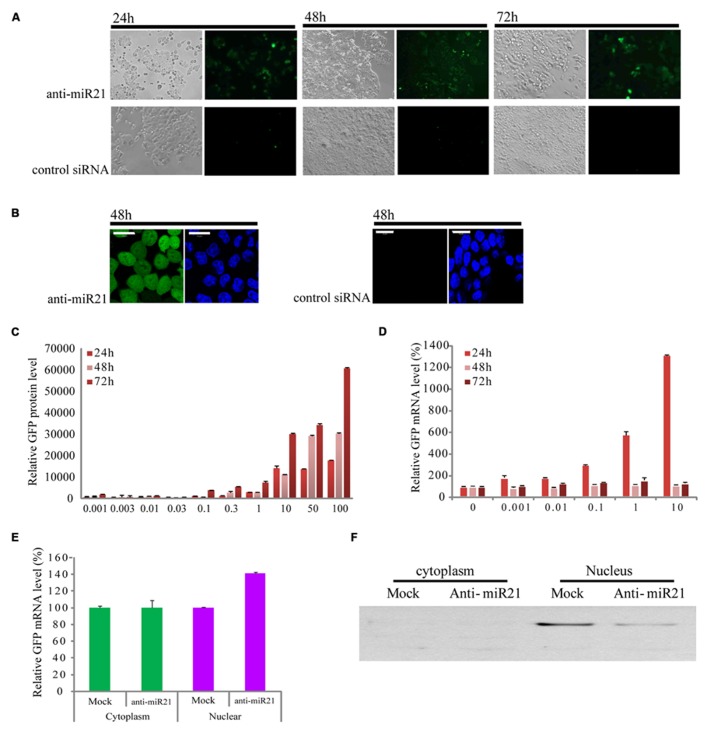
**Effect of anti-miR21 on miR21 activity. (A)** Bright field images of HCT116-GFP cells that were transfected with anti-miR21 or control siRNA and grown for the indicated times. **(B)** *left*, confocal microscopy image of HCT116-GFP cell 48 h after transfection with anti-miR21. Hoechst33342 stain (blue) indicates nuclei. *right*, confocal microscopy image of HCT116-GFP cell 48 h after transfection with control siRNA. **(C)** Relative GFP fluorescence of HCT116-GFP cells transfected with anti-miR21 (0.001–10 nM). Values represent mean ± SD (*n* = 3) from three independent experiments. **(D)** Quantitative GFP mRNA levels (normalized by GAPDH), determined by Q-PCR, in HCT116-GFP cells transfected with anti-miR21 (0.001–100 nM). **(E)** GFP mRNA levels in nuclear and cytoplasmic extracts of HCT116-GFP cells transfected with control siRNA and anti-miR21. MiRNA levels were determined as described in **(D)**. **(F)** Confirmation of separation of nuclear and cytoplasmic fractions. Aliquots containing 30 μg of total protein from cytoplasm and nuclear extracts were separated by 12% SDS-PAGE gel, transferred onto nitrocellulose membrane, and probed with antibodies specific for the nuclear protein fibrillarin.

In a previous study, we constructed a stable clonal CRC cell line that contains an integrated enhanced green fluorescent protein (*EGFP*) gene that has a fully complementary target site for miR21 in the 3′ UTR (HCT116-GFP) (Meister et al., 2004b; Castanotto et al., 2007). In this cell line, expression of *EGFP* is knocked down by the endogenous expression of miR21, which is up-regulated in CRC (Slaby et al., 2007; Schetter et al., 2008). HCT116-GFP cells transfected with anti-miR21 showed restored expression of GFP at 24, 48, and 72 h after transfection, but we did not detect GFP signal in cells transfected with control siRNAs (**Figure 1A**). To order to improve the image of the cell, we took the cell picture with confocal microscopy. The activation of GFP by anti-miR21 is shown in **Figure 1B**. These results confirmed that anti-miR21 can activate GFP translation by binding to miR21 to block binding of miR21 to the 3′ UTR of *EGFP* in CRC cells.

### ANTI-miR21 EFFECT OCCURS, WHICH ACTIVATES GFP mRNA EXPRESSION IN THE NUCLEUS

To determine whether the anti-miR21 could improve RNA and protein level which showed the post-transcriptional gene silencing (PTGS), we examined the expression of EGFP protein and mRNA in HCT116-GFP cells at 24, 48, and 72 h after transfection with a range of concentrations of anti-miR21 (0.001–100 nM; **Figures 1C,D**). Treatment with anti-miR21 increased the expression of *EGFP* protein in a dose- and time-dependent manner (**Figure 1C**). Surprisingly, a high level of expression of GFP mRNA was detected at 24 h (**Figure 1D**). To systematically determine where in the cell anti-miR21 inhibits miR21, nuclear and cytoplasmic fractions were isolated from HCT116-GFP cells transfected with the anti-miR21. Remarkably, expression of *EGFP* was increased in the nuclear fractions containing anti-miR21 (**Figure 1E**). The separation of cytoplasm and nuclear protein was confirmed by western blot analysis for the nuclear protein fibrillarin (**Figure 1F**). Recently Nishi et al. (2013) showed that miRNA-mediated gene silencing localizes to the nucleus. Our results suggest that the antagomir blocks miR21 in the cytoplasm and in the nucleus.

### ANTI-miR21 SLIGHTLY AFFECTS THE DEGRADATION OF ENDOGENOUS miR21

**FIGURE 2:**
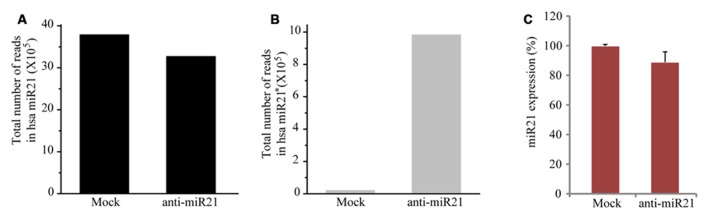
Expression level of miR21 and miR21^*^. Total counted number of deep-sequencing reads containing a corresponding sequence to miR21 **(A)** and miR21^*^. ^*^ indicates the small RNA processed from the hairpin arm opposite the mature miR21. **(B)** from HCT116-GFP cells transfected with anti-miR21 or mock control. **(C)** Quantitative miR21 expression determined by TaqMan probe Q-PCR of HCT116-GFP cells transfected with anti-miR21 or mock control. Values represent mean ± SD (*n* = 3) of three independent experiments.

To establish whether the anti-miR21 acts by affecting miR21 levels, we performed deep sequencing and TaqMan microRNA assay. When miR21 was inhibited using the anti-miR21, the number of total counts of sequences that matched miR21 reduced by 13% (**Figure 2A**). The levels of the complementary anti-miR21 sequence (miR21^*^) were dramatically increased in anti-miR21 treated cells (**Figure 2B**). To validate the results of the deep sequencing assay, we monitored the steady-state accumulation of mature miR21 through a fluorescent probe-coupled PCR assay (TaqMan probe qPCR). Similar to the sequencing data, levels of mature miR21 were 10% lower in anti-miR21-treated HCT116 cells after transfection compared to mock transfected cells (**Figure 2C**). These results indicate that the antagomir slightly contributed to the degradation of endogenous miR21 in HCT 116 cells.

### miRNAs ARE PERTURBED BY THE ANTI-miR21 ANTAGOMIR

**FIGURE 3:**
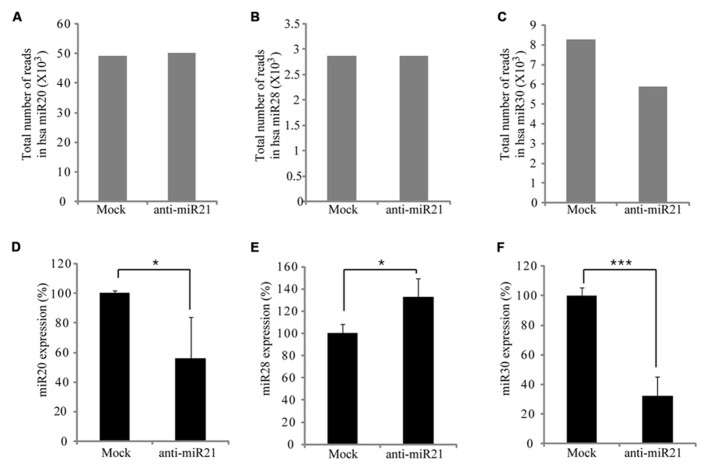
**Regulation of miRNA expression by anti-miR21 in CRC.** Expression levels of miRNAs that had the highest counts in HCT116 deep-sequencing results. Total number of RNA sequence reads for miR20 **(A)**, miR28 **(B)**, and miR30 **(C)** in HCT116-GFP cells transfected with anti-miR21 and mock control. Normalized expression levels of miR20 **(D)**, miR28 **(E)**, and miR30 **(F)** as determined by quantitative PCR using miRNA taqman probes. Error bars indicate SD. ^*^p < 0.05, ^***^p < 0.001.

Some miRNAs repress several positive regulators in a pathway, whereas others target both positive and negative regulators, to possibly buffer against minor physiological variations that can trigger much larger changes in the physiology of the cell (Khraiwesh et al., 2010). In CRC cells, this buffering role could mean that miR21 simultaneously targets the PTEN, PDCD4, Spry-1, and NF1B oncogenes. Furthermore, miRNAs can cooperate with each other to regulate one or more pathways, which increases the flexibility of regulation (Papagiannakopoulos et al., 2008). To determine whether the anti-miR21 could effectively perturb another miRNA, we analyzed the expression of several miRNAs in the deep sequencing results. This revealed that miR20, miR28 and miR30 were up-regulated in CRC cells that received mock (**Figures 3A–C**). Yang et al. (2009) and Volinia et al. (2006) identified that miR21, miR20, and miR30 were highly expressed in colon cancer. Interestingly, the expression of miR30 was significantly decreased in cells transfected with anti-miR21 (**Figure 3C**). Analysis of the expression of miR20, miR28, and miR30 using TaqMan qPCR (**Figures 3D–F**) revealed that the steady-state levels of mature miR30 were decreased in cells transfected with anti-miR21 (**Figure 3F**). The down-regulation of miR30 by anti-miR21 indicates that the anti-miR21 antagomir interferes with the stability of another miRNA.

### PERTURBATION OF miR30 BY THE ANTAGOMIR OCCURS IN THE CYTOPLASM

**FIGURE 4:**
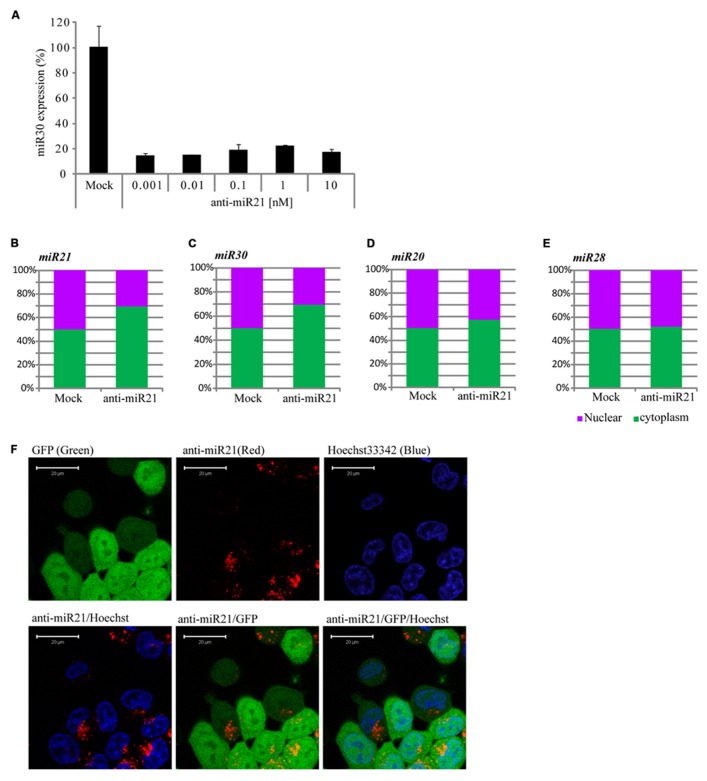
**Anti-miR21 changes the localization of miR30. (A)** Abundance of miR30 in HCT116-GFP cells transfected with anti-miR21 (0.01–10 nM), as measured by real-time qPCR. Cytoplasmic or nuclear localization of miR21 **(B)**, miR30 **(C)**, miR20 **(D)**, and miR28 **(E)** in cells transfected with anti-miR21 or mock control. **(F)** Confocal microscopy images showing the localization of transfected anti-miR21 (red). GFP is shown in green and Hoechst33342 in blue to indicate nuclei. The picture was taken by Zeiss LSM510 META 2-Photon microscope in Z-mode. Scale bars, 20 μm.

The siRNAs or miRNAs transfected in the cell affected the up-regulation of many mRNAs that were potentially targeted by miRNAs. Khan et al. (2009) suggested that transfection of small RNAs can reduce the endogenous miRNA function. To confirm the perturbation of miR30 by anti-miR21, we transfected HCT116 cells with a range of doses of anti-miR21 and measured miR30 expression by qPCR. When transfected at 0.001 nM, anti-miR21 led to an 80% reduction in the expression of miR30 (**Figure 4A**). Previous studies have shown that RISC components are localized at cytoplasmic foci of P-bodies that contain translationally repressed mRNAs. In P-bodies, translationally repressed mRNAs, which arise through PTGS, can remain in oligomeric structures for storage or can form complexes with cap-binding proteins and decapping enzymes, which triggers mRNA degradation. In other words, miRNAs in miRISC complexes could provide the sequence specificity for shuttling target mRNAs for storage or degradation in P-bodies (Kedersha et al., 2005; Liu et al., 2005; Valencia-Sanchez et al., 2006). We examined if mature miRNAs in CRC cells localized to the nucleus or cytoplasm (**Figures 4B–E**). miR20 and miR28, whose expression levels were not affected by anti-miR21 (**Figures 3A,B,D,E**), showed the same expression levels in the nucleus and cytoplasm in the presence or absence of anti-miR21 treatment (**Figures 4D,E**). In contrast, miR21 and miR30, which were regulated by anti-miR21, predominantly localized to the cytoplasm in cells treated with the antagomir (**Figures 4B,C**).

**FIGURE 5:**
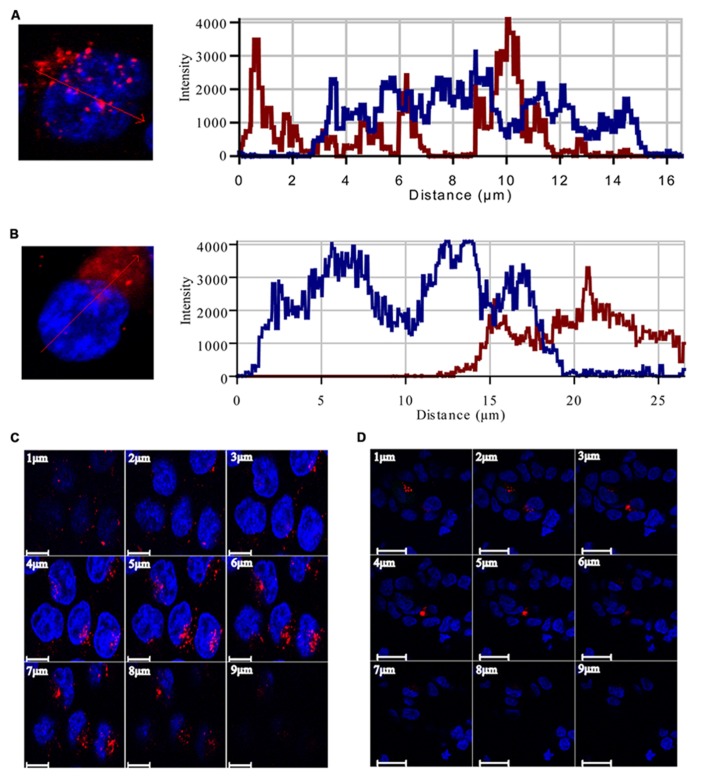
**Localization of anti-miR21 and pre-miR21.** Localization of Cy3-labeled anti-miR21 **(A)** or Cy3-labeled pre-miR21 **(B)** (both red). Nucleus indicated by DAPI (blue). The corresponding line scans were taken over the area indicated by the arrow. Projections of the Z scans are displayed for anti-miR21 **(C)** and pre-miR21 **(D)**. Scale bar, 20 μm. Z-scan; Depth, 9 μm.

To study the pattern of anti-miR21 localization in live cells, we used confocal microscopy to image HCT116-GFP cells transfected with Cy3-labeled anti-miR21. Anti-miR21 was largely seen in the cytoplasm, but we also detected it in the nucleus (**Figure 4F**). To verify the localization of anti-miR21 and pre-miR21 in the cytoplasm and nucleus we co-transfected HCT116 cells with Cy3-labeled pre-miR21 and anti-miR21; pre-miR21 can be processed to mature miR21 in cells, so the Cy3 signal would indicate the localization of miR21 in cells. Anti-miR21 strongly accumulated in both the nucleus and cytoplasm, as evidenced by the colocalization of Cy3-labeled anti-miR21 with the nuclear marker DAPI (**Figure 5A**). Cy3-labeled pre-miR21 was mostly present in the cytoplasm of HCT116 cells transfected with anti-miR21 (**Figure 5B**). **Figures 5C,D** of a nine-step Z-position sectional scanning demonstrated the localization of anti-miR21 and pre-miR21 in cytoplasm and nucleus. These results suggested that miR21 is also mostly present in the cytoplasm of HCT116 cells transfected with anti-miR21.

### ANTI-miR21 INDUCES INHIBITION OF ANGIOGENESIS

**FIGURE 6:**
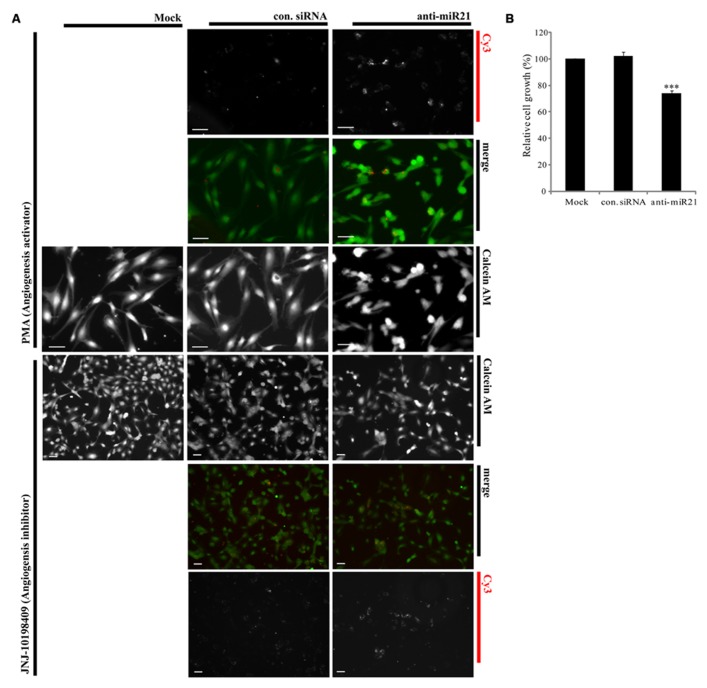
**Effect of anti-miR21 on angiogenesis and cell survival. (A)** Confocal microscopy images of tubular formation of HUVEC2 cells transfected with Cy3-labeled control siRNA or anti-miR21grown in the presence of an angiogenesis inhibitor (JNJ-10198409; 0.3 μM) or activator (PMA; 0.6 μM). In the merged image, Calcein AM is shown in green and Cy3-labeled anti-miR21 in red. The picture was taken by Zeiss AxioVert 200 inverted microscope. Scale bars, 50 μm **(B)** Relative cell growths of HCT116-GFP cells that were transiently transfected with negative control siRNA or anti-miR21 (5 nM). The cells were allowed to grow for 2 days before MTS assay. Values are means of four experiments ± SD. ^***^p < 0.005 versus control.

Overexpression of miR30 in endothelial cells leads to increased vessel number and length, and down-regulation of miR30 is associated with inhibition of angiogenic pathways (Birdsey et al., 2012). Both miR21 and miR30 are overexpressed in HUVECs (Heusschen et al., 2010). Using an *in vitro* angiogenesis assay, we saw a correlation between expression of anti-miR21 and angiogenesis in HUVECs. We transfected HUVECs with Cy3-labeled anti-miR21 and control siRNA, and then treated the cells with an angiogenesis activator or inhibitor. Angiogenesis activator Phorbol-12-myristate-13-acetate (PMA) enhanced the ability of HUVEC to organize into tubular networks by induced VEGF expression (Xu et al., 2008). Angiogenesis inhibitor JNJ-10198409[(6,7-dimethoxy-2,4-dihydro-indeno [1,2-c]pyrazol-3-yl)-(3-fluro-phenyl) -amine] inhibited tyrosine kinase activity of growth factor receptors such as the platelet-derived growth factor (PDGF-BB; D’Andrea et al., 2005). After 1 day of treatment with the activator or inhibitor, we stained cells with calcein AM, and analyzed their shape and detected the Cy3 label to determine the RNA localization (**Figure 6**). Cells failed in tube formation when treated with the angiogenesis inhibitor and transfected with control siRNA, mock or anti-miR21 (**Figure 6A**; lower area). However, HUVECs that were transfected with control siRNA or mock treatment did form tubular networks when treated with PMA, an angiogenesis-inducing agent. HUVECs transfected with anti-miR21 displayed a decreased propensity to form tubular networks of lesser length. This shape was similar to that of cells treated with the angiogenesis inhibitor JNJ-10198409 (**Figure 6A**; upper area). These results suggest that anti-miR21 down-regulated miR30, and this reduced miR30 expression affected inhibition of angiogenesis.

To confirm that anti-miR21 affects tumorigenesis in CRC we used the MTS assay to assess cell growth. Cell proliferation was reduced about 30% in anti-miR21-transfected cells as compared with cells transfected with control siRNA (**Figure 6B**). These results suggest that anti-miR21 influences cell growth in CRC.

### siRNAs CANNOT COMPETE WITH ANTI-miR21

**FIGURE 7:**
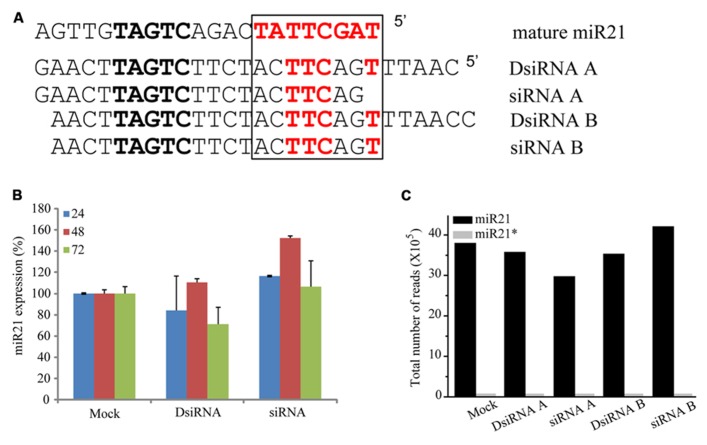
**siRNA did not controlled in miRNA. (A)** Seed sequence (black box) of with mature miR21 and the evaluated small RNAs. **(B)** Abundance of miR21 in HCT116 cells transfected with DsiRNA B or siRNA B, as measured by real-time qPCR. **(C)** Total number of reads corresponding to miR21 (black bar) or miR21^*^ (gray bar) in HCT116 cells transfected with DsiRNA A, siRNA A, DsiRNA B or siRNA **(B)**.

To determine if overexpressing other non-coding RNAs impaired the function of miR21, we transfected HCT116-GFP cells with small interfering miR21 RNAs (siRNAs; **Figure 7A**). Using TaqMan qPCR, we detected expression of mature miR21 in the transfected cells. Previous studies have shown that a 21mer short hairpin RNA (shRNA) blocks the action of miR21 but a 25mer shRNA does not (Grimm et al., 2006). In our system, neither the 21mer siRNA nor 25/27mer siRNAs affected miR21 expression (qPCR data in **Figure 7B** and deep sequencing data in **Figure 7C**). These results suggest that the siRNAs do not affect the mechanisms of miR21 transcription and processing or mRNA regulation.

## DISCUSSION

**FIGURE 8:**
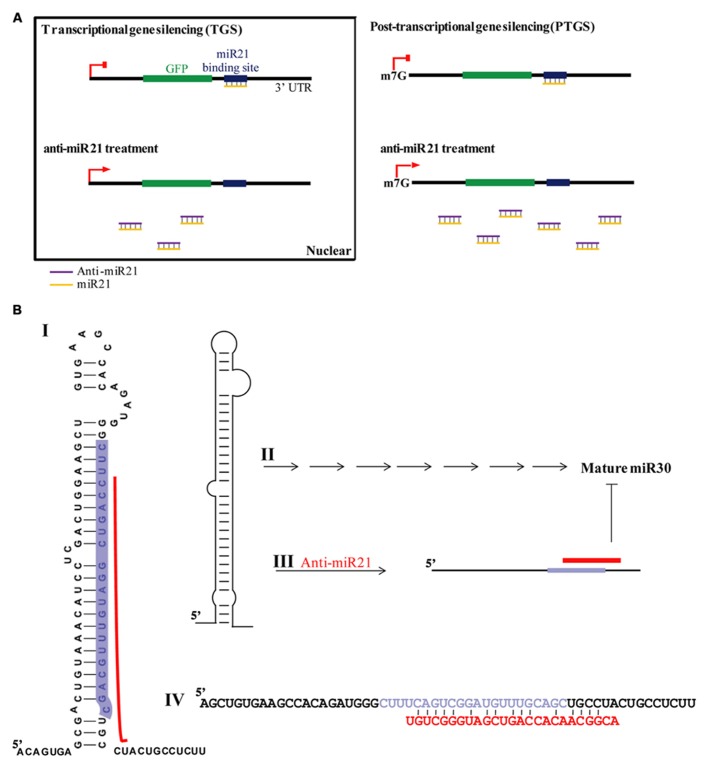
**Model for anti-miR21 activity. (A)** TGS and PTGS models for mechanism by which anti-miR21 inhibits miR21. **(B)** Model for mechanism by which anti-miR21 inhibits miR30. Anti-miR21 blocks miR30 on the basis of matching sequence. I. The structure of pri-miR30. Red bar indicates hybrid area of anti-miR21, as identified by CLC Bio Main Workbench software. Highlighted sequence is mature miR30. II. Pri-miR30 is normally processed into mature miR30 through the normal miRNA mechanism. III. The structure of pri-miR30 can be changed by anti-miR21. IV. Sequence alignment of pri-miR30 (upper sequence) with anti-miR21 (red sequence). Gray sequence shows the mature miR30.

The emerging significance of miRNAs in cancer has spiked major interest for possible use in cancer therapy, resulting in many cancer-profiling studies of miRNAs. miR21 is greatly over-expressed in CR colon cancer cells (Ambros, 2004), used target of conventional cancer therapeutics and biomarker (Kloosterman and Plasterk, 2006; Bushati and Cohen, 2007). Inhibition of miR21 function by anti-miR21 has become an important and widely used approach in therapeutic modalities of colon cancer therapy. It is thought that the predominant function of miRNAs is translational repression of target mRNAs (PTGS). A model of RNA silencing suggests that the RISC complex can guide target RNA cleavage or translational repression depending on the extent of sequence complementarity between the miRNA and its target (Meister et al., 2004a; Yi et al., 2005; Grimm et al., 2006; Castanotto et al., 2007). We have established that anti-miR21 regulates miR21 in the nucleus by transcriptional gene silencing (TGS). This is consistent with mounting evidence that miRNAs play important roles in the nucleus (Hwang et al., 2007; Guang et al., 2008; Kim et al., 2008; Marcon et al., 2008; Ohrt et al., 2008; Politz et al., 2009; Nishi et al., 2013) and suggests that miR21 could lead to an effect on gene silencing in the nucleus, and anti-miR21 can inhibit this mechanism in nucleus. Consistent with our observation, it has been shown that activation of GFP RNA by anti-miR21 is caused in the nucleus (**Figure 1E**). We also demonstrated that more anti-miR21 was present in the cytoplasm than in the nucleus (**Figures 4E** and **5**), suggesting there is not much need for miR21 to have lifted GFP down-regulation by anti-miR21 in the nucleus (**Figure 8A**). Therefore, we hypothesize that anti-miR21 is not only regulated at the post-transcriptional level, but is also subject to fine-tuning by a TGS mechanism. The potential role of nuclear anti-miR21 in the regulation of gene transcription by inhibition of miR21 expose a totally new and exciting application for cancer therapy of small non-coding RANs.

It has been proposed that perturbation of miRNAs can cause inference of miRNA regulatory network in cancer (Lujambio and Lowe, 2012). Thus, perturbed levels of many miRNAs after antagomir treatment can change the expression of many genes that are involved in transcriptional repression, leading to unexpected outcomes. In principle, miRNAs repress several positive components of a pathway, whereas others target both up- and down- regulation, possibly to change against targeted mRNAs in cytoplasm that could trigger much larger changes in the cells. Featured in this study is that reduced levels of miR30 by antagomir of anti-miR21 can cause down-regulation of the miRNA-targeted gene, which in turn can lead to unexpected effects. Indeed, anti-miR21 led to decreased miR30 expression level, inhibiting tubular forming ability *in vitro* (**Figure 6A**). Recent study is identifying DLL4 as a target of miR30 and to demonstrate a key role for miR30 in angiogenesis. The miR30 regulates angiogenesis through the highly conserved, molecular targeting of DLL4 (Bridge et al., 2012). This, together with decreasing the expression of miR30 and miR21 in endothelial cells, reduced the formation of cell networks *in vitro*. Moreover, down-regulation of miR21 in colon cancer cells leads to inhibition of cell proliferation (**Figure 6B**). These results suggest that anti-miR21 inhibited oncogenesis by blocking the activity of miR21 and regulated angiogenesis, which is critical for supporting tumor growth, by perturbing miR30.

Our data indicate that anti-miR21 led to decreased miR30 expression level, inhibited angiogenesis *in vitro*. Several possibilities exist for this situation. One of this is small RNA perturbation with small RNA competition for RISC complex. Transfection with small RNAs or miRNAs can disturb the function of endogenous miRNA, probably by saturating the RISC pathway (Khan et al., 2009). For example, exogenous small RNAs can compete for endogenous RISC components required for miRNA processing (Liang et al., 2013). A another study reported that small RNAs can quickly lead to a reduction of miRNA levels by competition for RISC, leading to disassembly of miRNAs form RISC, and ultimately causing reduction of steady state levels of miRNAs (Pan et al., 2011). Indeed, in our study, we found that miR30 was down-regulated upon anti-miR21 treatment, as determined by deep-sequencing and miRNA qPCR (**Figures 3C,F**). Our results indicate that perturbation of miR30 could occur even at low anti-miR21 concentration, such as 0.001 nM (**Figure 4A**). The inhibition of miR21 and miR30 upon anti-miR21 transfection preferentially occurred in the cytoplasm (**Figures 4B,C**). This finding suggested that endogenous miRNAs may be replaced within the RISC complex by exogenous antagomir because of differing affinities for RISC components, or because of competition between the miRNA and antagomir for the RISC machinery. Another possibility is sequence homology between anti-miR21 and miR30. To investigate how anti-miR21 regulates the expression level of miR30, we answered the question to validate target predictions of anti-miR21 for pri-miR30, and we aligned the sequences of anti-miR21 and pri-miR30 (**Figure 8B**). Surprisingly, the sequences of anti-miR21 mostly matched the 3′ end of pri-miR30, in line with the known effects of miRNA perturbation on knockout of PTGS (Pan et al., 2011; Liang et al., 2013), We also noticed that anti-miR21 is down-regulated in miR30, suggesting that anti-miR21 may bind to pri-miR30, and that this binding may disrupt the secondary structure of pri-miR30 and prevent miRNA processing downstream.

In conclusion, the results of our current investigation suggest that anti-miR21 is an effective therapeutic strategy for colon cancer by regulating miR21 pathway in nucleus and inhibiting angiogenesis regulated anti-miR21 as perturbing miR30. Therefore, this study has validated anti-miR21 as a target of miR30 and demonstrated a role for anti-miR21 in perturbing cell proliferation and angiogenesis, two critical features of oncogenesis.

## Conflict of Interest Statement

The authors declare that the research was conducted in the absence of any commercial or financial relationships that could be construed as a potential conflict of interest.
